# Spittlebugs produce foam as a thermoregulatory adaptation

**DOI:** 10.1038/s41598-018-23031-z

**Published:** 2018-03-16

**Authors:** Mateus Tonelli, Guilherme Gomes, Weliton D. Silva, Nathália T. C. Magri, Durval M. Vieira, Claudio L. Aguiar, José Maurício S. Bento

**Affiliations:** 1 Department of Entomology and Acarology, University of São Paulo, Piracicaba, SP Brazil; 2 Department of Physics and Interdisciplinary Science, University of São Paulo, São Carlos, SP Brazil; 3 Hugot Sugar Technology Laboratory, University of São Paulo, Piracicaba, SP Brazil; 4 Erythro Assessoria Química S/C Ltda., Campinas, SP Brazil

## Abstract

Insects have evolved multiple mechanisms to adapt to variations in environmental temperatures, including postural control of solar input, variations in diurnal activity, external morphological structures and selecting/generating microhabitats. Foam produced by *Mahanarva fimbriolata* nymphs (also known as root spittlebugs) was found to aid in creating a constant thermal microhabitat despite environmental temperature fluctuations. The temperature within the foam was found to be similar to that of soil during the day and remained constant despite fluctuating external temperatures. In chemically analysing the composition of the foam, palmitic and stearic acids, carbohydrates and proteins were detected. These substances have previously been shown to act as a surfactant to stabilize and modulate foams. Since the immature ancestor of the spittlebug developed below ground, it is speculated that the foam may function as an ‘extension’ of the soil and, thus, may have enabled the spittlebug to emerge from the soil and adopt an epigean lifestyle.

## Introduction

As insects are ectothermic (i.e., the internal temperature of the body varies according to the air temperature), they have evolved different mechanisms to regulate body temperature^1^. One common adaptation for thermoregulation is the creation of microhabitats. For example, eusocial hymenopterans and termites build elaborate nests to reduce the stress caused by environmental temperature fluctuations^2,3^. Cicada nymphs build below ground tunnels that allow them to live for years under favourable thermal conditions^4^. In contrast, nymphs of spittlebugs, a group that is phylogenetically closely related to cicadas, can be found below the soil surface^5,6^, at ground level^7^ or even far above the soil surface^8^. How they maintain a constant/suitable body temperature without the protection of ground insulation has not been previously reported.

Phylogenetic studies have shown that the first spittlebugs evolved approximately 200 million years ago from an ancestor in which nymphs developed below ground^7,9,10^. However, unlike the closely related cicadas, the front legs of spittlebug nymphs are not strong enough to burrow into the soil^9^. One potential mechanism that has been proposed for thermoregulation in spittlebugs is the foam that they produce and cover themselves with (commonly referred to as ‘cuckoo spit’)^11,12^. The nymphs produce foam by sucking air into the ventral cavity of their abdomen, that is then trapped in the fluid of the Malpighian tubules, resulting in the creation of bubbles in the terminal part of the abdomen^12,13^. The foam comprises liquid, air, and surface-active molecules that reduce surface and interfacial tension to form emulsions^11,14^. The liquid in the foam is derived from the plant sap upon which the nymphs feed^15^.

Interestingly, some amphibians, such as frogs, produce foam that protects their eggs and embryos against predation and desiccation while maintaining temperature and oxygen at appropriate levels^16–18^. Even though a similar function has been proposed for spittlebug foam^19,20^, experimental evidence of thermoregulation by their foam has not been shown.

The chemical composition allowing for a rigid bubble architecture in spittlebug foam is poorly understood^14^. Identifying the biochemical components may provide insight into the capacity of foam to contribute to thermoregulation. Proteins, carbohydrates and lipids can stabilize foam^21–24^, however little is known about the presence and quantity of these substances in spittlebug foam.

*Mahanarva fimbriolata* are spittlebugs that feed on sugarcane roots^25^. These cercopids develop on the exposed roots on soil surface or below ground^5^ and form a distinctive foam when in the nymph stage. Here we examine the role that foam may play in thermoregulation for spittlebug nymphs.

## Results

### Foam as a thermal microhabitat

**Figure 1:**
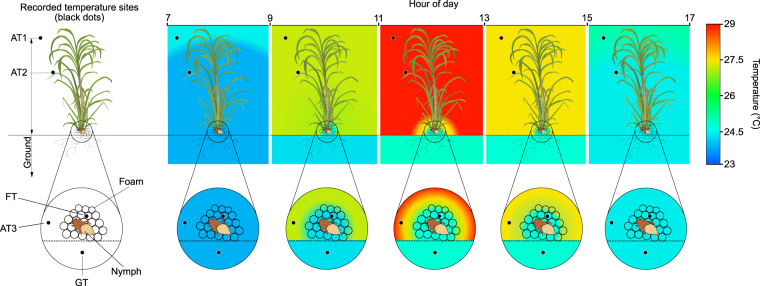
Thermal variation in the foam produced by *Mahanarva fimbriolata* nymphs. Temperature variation in the foam produced by *M. fimbriolata* nymphs and in their environmental surroundings during a hot summer’s day in a sugarcane field in Piracicaba, São Paulo, Brazil. Different colours indicate significant differences between recorded temperature sites within the same sampling time, according to one-way ANOVA followed by a Bonferroni post hoc test (*P* < 0.05) (*n = *25). AT1 = air temperature at 2.5 m above ground; AT2 = air temperature at 1.5 m above ground; AT3 = air temperature at 0.1 m from foam and ground; FT = temperature inside foam; GT = temperature 0.1 m below ground.

**Figure 2:**
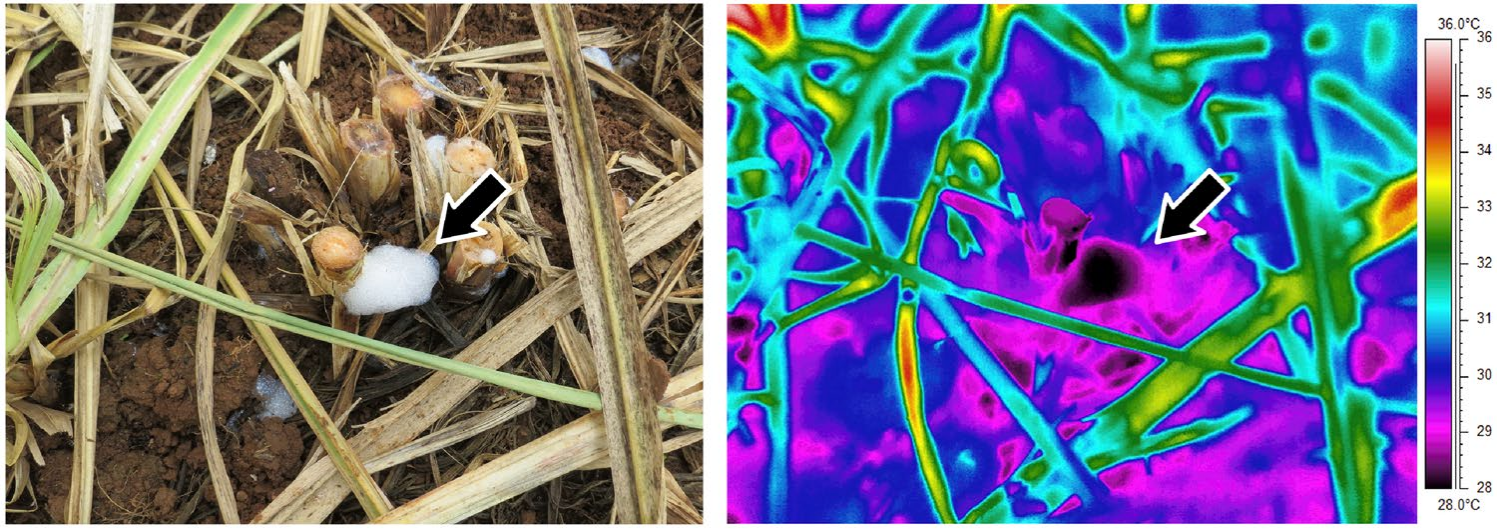
The foam promotes insect thermal protection. Conventional and corresponding infrared photographs of the foam produced by *Mahanarva fimbriolata* nymphs and of their surroundings in a sugarcane field in Piracicaba, São Paulo, Brazil, at 13h00 on a hot summer’s day. The photographs show the importance of the foam to maintaining the microhabitat temperature lower than the surrounding temperature.

To determine whether the internal temperature of spittlebug foam changes with fluctuations in external temperature, several local temperatures were monitored including: outside the foam, ground temperature near the foam and inside the foam. These temperature recordings were performed during summer in a sugarcane field where nymphs reside. While external temperatures range from 24.43 ± 0.44 °C to 29.20 ± 1.66 °C, the internal foam temperature was found to vary to a much smaller degree (Fig. 1). In the middle of the day (11h00–13h00) when the external temperature was 29.20 ± 1.66 °C, the foam temperature was observed to be significantly lower at a temperature of 25.18 ± 0.63 °C (Fig. 1, one-way ANOVA followed by a Bonferroni post hoc test, *n = *25, F_(1,100)_ = 88.763, *P < *0.0001) (Detailed values of means ± SD corresponding to Fig. 1 are shown in Supplementary Table S1). Indeed, despite fluctuating external temperatures, a uniform foam temperature was observed throughout the day (i.e. 25 ± 0.78 °C (mean ± SD)). Specifically a previous investigation found that 25 °C resulted in the greatest nymph viability^26^. In monitoring over 10 hours during the day, the surface temperature difference between the foam and the soil was ≤ 0.2 °C while the maximum difference between foam and the external temperature was ≥ 4.0 °C. This indicates that the temperature of the soil and the foam are similar (Fig. 1, one-way ANOVA followed by a Bonferroni post hoc test, *n = *25, F_(1,100)_ = 0.008, *P = *0.928). Using thermograms obtained from an infrared camera, the difference in temperature near the foam can be visualized (Fig. 2).

**Figure 3:**
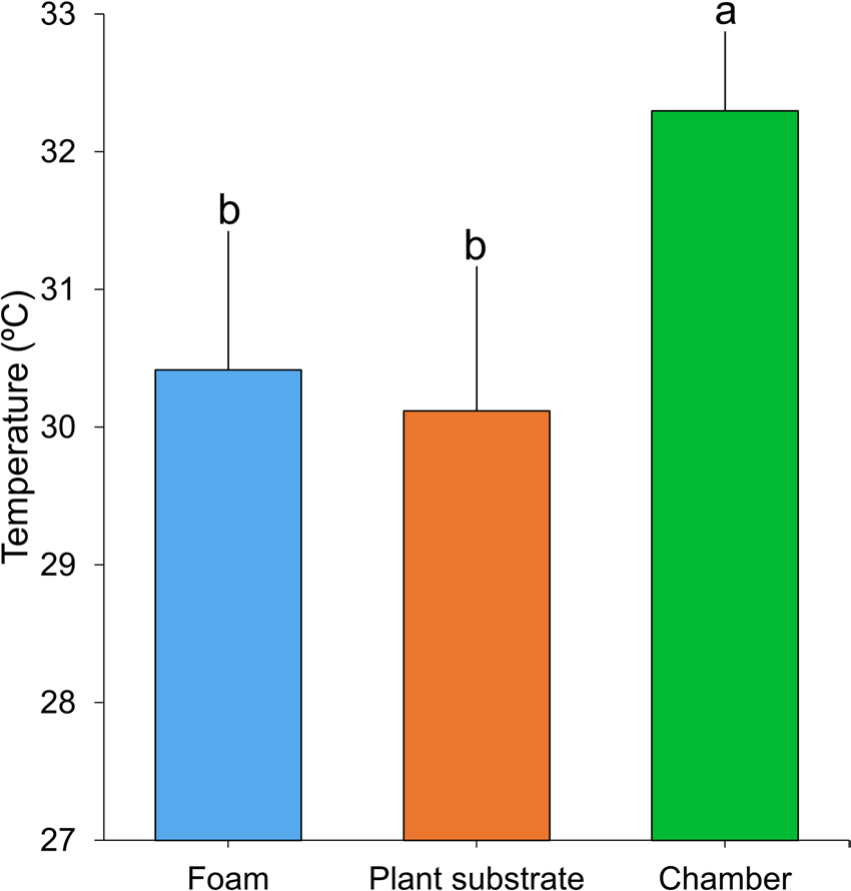
The temperature of spittlebug foam under controlled conditions. Comparison of the temperature of the foam produced by *Mahanarva fimbriolata*, plant substrate (soil) and surrounding air temperature in a growth chamber. Bars represent the temperature mean ± SD. Bars with different letters are significantly different according to one-way ANOVA followed by a Bonferroni post hoc test (*P* < 0.05) (*n* = 25).

To more rigorously examine the thermocapacity of the foam, nymphs were evaluated in a fitotron in which the temperature was controlled and elevated above normal field conditions. When the fitotron temperature was raised to 32.29 ± 0.58 °C (mean ± SD) for 30 minutes, foam temperature remained at 30.41 ± 1.01 °C (mean ± SD) which is approximately 2 °C below the air temperature (Fig. 3, one-way ANOVA followed by a Bonferroni post hoc test, *n = *25, F_(1,25)_ = 57.220, *P < *0.0001). These results indicate that the foam acts as a thermoregulator at 32 °C, which has previously been shown to be lethal for nymphs^26^. The combined field and laboratory data indicate that nymph foam production (Supplementary Movie 1) results in a relatively constant internal temperature at a wide range of external air temperatures that creates a suitable thermal microhabitat for nymph survival.

### Foam chemical composition

Using gas chromatography-flame ionization detection (GC-FID) and gas chromatography-mass spectrometry (GC-MS) two major peaks were identified as palmitic acid and stearic acid; co-injection with commercial standards confirmed their identity. Amounts of palmitic and stearic acid in the foam were 2.54 ± 0.88 and 2.78 ± 0.97 µg ml^−1^ foam (means ± SE), respectively. Total carbohydrates were quantified by means of the phenol-sulfuric acid method^27^, with 0.579 ± 0.05 µg ml^–1^ foam (mean ± SE). Based on the Bradford method^28^ total protein was 320 ± 50 µg ml^–1^ foam. These components are recognized as important substances in the formation and stabilization of foam bubbles^29–32^, and as such are likely necessary for maintaining a stable bubble layer around nymphs.

## Discussion

Insects have evolved complex mechanisms to regulate their body temperature within a remarkably narrow range for successful survival and reproduction^33–35^. Here, we showed that *M. fimbriolata* nymphs produce and cover themselves with foam as a thermoregulatory adaptation that enable spittlebugs to maintain their body temperature within the optimal range for development^26^. The temperature measured inside the foam was similar to the soil even though the air temperature in both the field and fitotron varied to a much greater extent (Figs 1 and 3). Since spittlebug and some below ground insects are thought to share a common ancestor^7,9^, insect-produced foam may serves as an ‘extension’ of the soil and enable immature spittlebugs to exploit food sources for above ground feeding. Without such a domestic protection, delicate cuticles would leave spittlebug nymphs vulnerable to adverse abiotic epigean environmental factors, such as high temperature and low humidity^19^.

Lipids, carbohydrates, and proteins were detected in *M. fimbriolata* foam similar to the foam composition of other spittlebug species^11,14,15,36,37^. Proteins were detected in most foams analysed^11,14,15,36^ whereas lipids are much less common being observed only in the foam of Japanese spittlebugs^37^. While the chemical analysis has not been shown to directly provide thermal protection, lipids, carbohydrates and proteins that are present in spittlebug foam has previously been shown to function as a surfactant to stabilize the foam thereby reducing surface tension and modulating the size and distribution of bubble^29–31^. Lipids, including palmitic and stearic acids, are critical for the formation and stability of foams because of their elastic forces^32^. For example, palmitic, linolenic and pentadecanoic acid have been positively correlated with the height of cider-type beverage foams^24^. Proteins are involved, especially in the formation of film that reduce interfacial tension and increase the viscosity and elasticity of a foam^38–40^, which allows the foam to breathe and secure around the insect. Although carbohydrates have no direct effect on the air−water interface, they promote interactions among proteins, which create a stable film that stiffens and stabilizes the foam^41^.

While the mechanism behind the observed thermal protection is proposed to be due to thermal insulation the extent in which evaporative cooling may play a role in controlled temperature conditions still needs to be investigated. Evaporative cooling has been demonstrated for hemipterans that feed on xylem and do not produce foam, such as the cicada *Okanagodes gracilis*, that regulate their body temperature by water loss through pores in the dorsal thorax and abdomen^33^. Interestingly, for foam-producing *Aphrophora saratoga* nymphs, water evaporation from foam was demonstrated be lower than from free-water surface, making the foam an uncertain protection against desiccation for this species^42^. In future studies, by measuring temperature of dried cercopid foam differentiating these two mechanisms experimentally should be possible.

In summary, nymph-produced foam forms a microhabitat for the thermoregulation of *M. fimbriolata* nymphs. Future investigations on physical properties of the foam, especially optical reflection and heat dissipation will provide further insights into the phenomenon reported here.

## Methods

### Thermal microhabitat

To determine whether the foam covering *M. fimbriolata* nymphs has a thermoregulatory role, we conducted a field bioassay during the summer of 2015 in a sugarcane field in Piracicaba, São Paulo, Brazil (22°42′06″S, 047°33′50″W). The sugarcane plants were approximately 2 m tall, with 1 m between rows. We selected 25 sites inhabited by foam-covered fourth- and fifth-instar nymphs of *M. fimbriolata*, maintaining a minimum distance of 10 m between each site. Using a type K thermocouple (RDXL4SD, Omega Engineering, Stamford, CT, USA), we measured the temperature inside the foam, 0.1 m from foam and ground, 2.5 m and 1.5 m above ground, and 0.1 m below ground level at five time intervals that represented the natural variation in temperature during the day: 07h00–09h00, 09h00–11h00, 11h00–13h00, 13h00–15h00 and 15h00–17h00 (Fig. 1). We constructed a thermogram using an infrared camera (SC640 FLIR Systems, Boston, MA, USA) and analysed the temperature of the thermographic images using the ThermaCAM Researcher 2.9 software (FLIR Systems, Boston, MA, USA).

To investigate if the thermoregulation occurs at higher environmental temperatures than those achieved in the field experiments, we also evaluated the thermophysiology of the foam under controlled conditions using a fitotron growth chamber (ELETROLAB, São Paulo, SP, Brazil). Fourth and fifth-instar nymphs of *M. fimbriolata* were collected from the same field and carefully transferred to the roots of sugarcane plants aged 25–30 days growing in pots (200 ml) containing organic substrate (Golden-Mix, Ananindeua, PA, Brazil), with one nymph per plant, for a total of 25 replicates. The plants and insects were initially equilibrated at room conditions (25 ± 2 °C, 70 ± 10% UR) for 30 min before nymphs began to produce foam. Next, they were arranged within the growth chamber at a temperature of approximately 32 ± 0.11 °C. After 30 min of acclimation and temperature stabilization, we recorded the temperatures in the chamber, 1 cm below the surface of the soil in the pots, and inside the foam using the type K thermocouple.

### Fatty acid analysis

To analyse the fatty acids present in the foam, five foam samples were collected in the same sugarcane field cited above, placed in glass vials (10 ml) using a glass pipette and stored at −30 °C until analysis. Following an extraction sequence, 1 ml of each sample was derivatised through the application of ethyl chloroformate^43^. At the end of the derivatisation process, each sample was adjusted to 0.5 ml with cyclohexane as the solvent. Each sample received 5 μL of octacosane (internal standard solution at 1000 ng µL^−1^) (Sigma-Aldrich, St Louis, MO, USA). The derivatised samples were initially analysed by gas chromatography-flame ionization detection (GC-FID, Shimadzu GC-2010, Kyoto, Japan) using an HP-1 capillary column (Agilent Scientific, Santa Clara, CA, USA; 30 m × 0.25 mm × 0.25 µm). A 1 µL aliquot of each sample was injected in the splitless mode with an injector temperature of 240 °C using helium as the carrier gas. The column temperature was held at 60 °C for 1 min and then increased to 320 °C (15 °C min^−1^) and held for 10 min. The extract with the best resolution was reanalysed with a gas chromatograph coupled to a mass spectrometer (GC-MS, Varian 4000, Palo Alto, CA, USA) using an HP5-MS column (JeW Scientific, Folsom, CA, USA; 30 m × 0.25 mm × 0.25 µm) and helium as the carrier gas. Injection (1 µL aliquot) was conducted in the splitless mode, and the column temperature programme was the same as that described for the GC-FID procedure above. The two major peaks were identified by comparing their mass spectra with those of the NIST 98 library and confirmed by co-injecting the authentic standards (Sigma-Aldrich, St Louis, MO, USA) with the sample. Amounts were estimated based on the peak area relative to the amount of internal standard (octacosane) and corrected according to the volume of foam used for the derivatisation.

### Carbohydrate analysis

Total carbohydrate was estimated using the phenol-sulfuric acid method^27^. Briefly, five samples of foam produced by *M. fimbriolata* nymphs were collected, and an aliquot of 1 ml of each sample was vortexed with 25 µL of phenol (80% m/v) and 2.5 ml of sulfuric acid. The resulting mixture was allowed to stand for 20 min and then vortexed again. For the control, we used 1 ml of distilled water and followed the same steps as above. The absorbance of the mixed samples was measured spectrophotometrically using a UV mini 1240 Shimadzu (Shimadzu, Tokyo, Japan) at a wavelength of 490 nm. The total concentration was calculated based on a standard curve using glucose (0.01 mg L^−1^) in the range of 100–1000 µg.

### Protein analysis

Total protein present in the foam was quantified according to the Bradford technique^28^. Five samples of foam were collected in the same sugarcane field and 50 µL of each sample was dissolved in 3.95 ml of Milli-Q water, and 1 ml of Bradford reagent (Coomassie Brilliant Blue G with phosphoric acid and methanol) was added. The absorbance of the samples was measured using a spectrophotometer UV mini 1240 Shimadzu (Shimadzu, Tokyo, Japan) at a wavelength of 595 nm. As a control, we used 4 ml of Milli-Q water and 1 ml of protein reagent. The total protein concentration in the foam was calculated based on a standard curve for bovine serum albumin (BSA) at intervals of 0.02 to 0.3 mg.

### Statistical analysis

The normality and homogeneity of the temperatures recorded in the field observations and in the laboratory assay were analysed using Kolmogorov-Smirnov and Bartlett tests. To limit the experiment-wise error rates to acceptable level in multiple comparisons with a low numbers of related groups, means the temperatures of the foam and those of other recorded sites were compared using one-way ANOVA followed by a Bonferroni post hoc test (P < 0.05)^44^. All analyses were performed using the SAS statistical software^45^.

### Data availability

The data that support the findings of this study are available from the corresponding author (J.M.S.B.) upon reasonable request.

## Electronic supplementary material

Supplementary Movie

Supplementary Table S1
