# Supplementary material for: Spittlebugs produce foam as a thermoregulatory adaptation
Source: Sci Rep. 2018 Mar 16;8:4729. doi: 10.1038/s41598-018-23031-z (PMC5856825; doi:10.1038/s41598-018-23031-z)
Supplement: Supplementary file 2 — Supplementary Table S1 [file 41598_2018_23031_MOESM2_ESM.docx]

**Spittlebugs produce foam as a thermoregulatory adaptation**

Mateus Tonelli^1^, Guilherme Gomes^2^, Weliton D. Silva^1^, Nathália T. C. Magri^3^, Durval M. Vieira^4^, Claudio L. Aguiar^3^ & José Maurício S. Bento^1*^

^1^ Department of Entomology and Acarology, University of São Paulo, Piracicaba, SP, Brazil

^2^ Department of Physics and Interdisciplinary Science, University of São Paulo, São Carlos, SP, Brazil

^3^ Hugot Sugar Technology Laboratory, University of São Paulo, Piracicaba, SP, Brazil

^4^ Erythro Assessoria Química S/C Ltda., Campinas, SP, Brazil

^*^Correspondence and requests for materials should be addressed to J.M.S.B. (email: [jmsbento@usp.br](mailto:jmsbento@usp.br))

**SUPPLEMENTARY INFORMATION**

**Supplementary Table S1**: Temperature (°C) variation (mean ± SD) in the foam produced by *Mahanarva fimbriolata* nymphs and the environmental surroundings during a summer day in Piracicaba, São Paulo, Brazil. Different letters indicate significant difference between sampled sites within each time interval, based on a one-way ANOVA followed by a Bonferroni post hoc test (*P* < 0.05) (*n* = 25).

| **Recorded temperature sites** | **Hour of day** | | | | |
| --- | --- | --- | --- | --- | --- |
|  | **07h00–09h00** | **09h00–11h00** | **11h00–13h00** | **13h00–15h00** | **15h00–17h00** |
| **AT1** | 24.43 ± 0.44 a | 27.13 ± 1.04 a | 29.20 ± 1.66 a | 27.54 ± 0.82 a | 25.28 ± 0.68 a |
| **AT2** | 24.09 ± 0.35 b | 26.87 ± 1.00 a | 28.67 ± 1.55 a | 27.86 ± 0.69 a | 25.23 ± 0.59 a |
| **AT3** | 23.94 ± 0.24 b | 26.47 ± 0.92 a | 27.67 ± 0.94 b | 27.61 ± 0.59 a | 25.38 ± 0.52 a |
| **FT** | 23.56 ± 0.29 b | 24.64 ± 0.66 b | 25.18 ± 0.63 c | 25.24 ± 0.47 b | 24.57 ± 0.36 b |
| **GT** | 23.57 ± 0.24 b | 24.51 ± 0.76 b | 25.15 ± 0.61 c | 25.13 ± 0.49 b | 24.86 ± 0.44 b |

**AT1** = air temperature at 2.5 m above ground; **AT2** = air temperature at 1.5 m above ground; **AT3** = air temperature at 0.1 m from foam and ground; **FT** = temperature inside foam; **GT** = temperature 0.1 m below ground.
